# A case report and literature review of myocardial infarction with nonobstructive coronary arteries (MINOCA) possibly due to acute coronary vasospasm induced by misoprostol

**DOI:** 10.3389/fcvm.2023.1115358

**Published:** 2023-05-26

**Authors:** Nguyen Viet Hau, Luu Thi Kim Han, Le Huu Nhat Minh, Nguyen Anh Kiet, Tang Tuan Phong, Nguyen Khanh Duong, Phan Thi Hoang Yen, Nguyen Xuan Vinh, Nguyen Quan Nhu Hao, Nguyen Nguyen, Thien Tan Tri Tai Truyen, Nguyen Quoc Khanh Le

**Affiliations:** ^1^ Emergency Department, University Medical Center, Ho Chi Minh City, Vietnam; ^2^ International Ph.D. Program in Medicine, College of Medicine, Taipei Medical University, Taipei, Taiwan; ^3^ AIBioMed Research Group, Taipei Medical University, Taipei, Taiwan; ^4^ Research Center for Artificial Intelligence in Medicine, Taipei Medical University, Taipei, Taiwan; ^5^ Pharmacy Department, University Medical Center, Ho Chi Minh City, Vietnam; ^6^ Department of Internal Medicine, Palmetto General Hospital, Hialeah, FL, United States; ^7^ Faculty of Medicine, Nam Can Tho University, Can Tho, Vietnam; ^8^ Professional Master Program in Artificial Intelligence in Medicine, College of Medicine, Taipei Medical University, Taipei, Taiwan; ^9^ Translational Imaging Research Center, Taipei Medical University Hospital, Taipei, Taiwan

**Keywords:** MINOCA, myocardial infarction in the absence of obstructive coronary artery disease, acute myocardial infarction, prostaglandin e1 analogue, coronary vasospasm.

## Abstract

Coronary artery vasospasm (CVS), an uncommon cause of acute chest pain, can be provoked by vasoconstriction-induced medications. Misoprostol, a prostaglandin analog, is a safe medication to terminate a pregnancy. However, misoprostol can cause coronary artery vasospasm due to vasoconstrictor properties, leading to acute myocardial infarction with nonobstructive coronary arteries (MINOCA), especially in patients with a high risk for cardiovascular disease. We report a case of a 42-year-old female with a past medical history of hypertension who presented with ST-elevation myocardial infarction following the administration of a high-dose Misoprostol. The fact that coronary angiogram and intravascular ultrasound revealed normal coronary arteries suggested transient coronary vasospasm. CVS is a severe but rare cardiac adverse effect associated with high-dose misoprostol. This medication should be prescribed with caution and close monitoring, especially in those with pre-existing heart disease or cardiovascular risk factors. Our case raises awareness of severe cardiovascular complications that can be related to using misoprostol in high-risk patients.

## Background

Coronary arteries disease without significant flow-limiting lesions or severe coronary vasospasm can result in an acute imbalance between oxygen demand and supply, causing acute myocardial infarction with non-obstructive coronary disease (MINOCA) (1). In 2019, the American Heart Association's practical guidelines and algorithm were established to provide clinical guidance for the diagnosis and treatment of MINOCA (2). Misoprostol, a prostaglandin E1 analog, is currently used under the recommendation of the National Institute For Health and Care Excellence 2019 and the American College of Obstetricians and Gynecologists 2020 for the termination of pregnancy (3, 4). However, misoprostol uncommonly can induce acute coronary vasoconstriction when used with a high dose (5). Herein, we report a case of a 42-year-old female suffering from acute STEMI due to severe coronary vasospasm following the administration of high-dose misoprostol (400–1200 mcg within 24–48 h) for abortion induction.

## Case presentation

**Figure 1:**
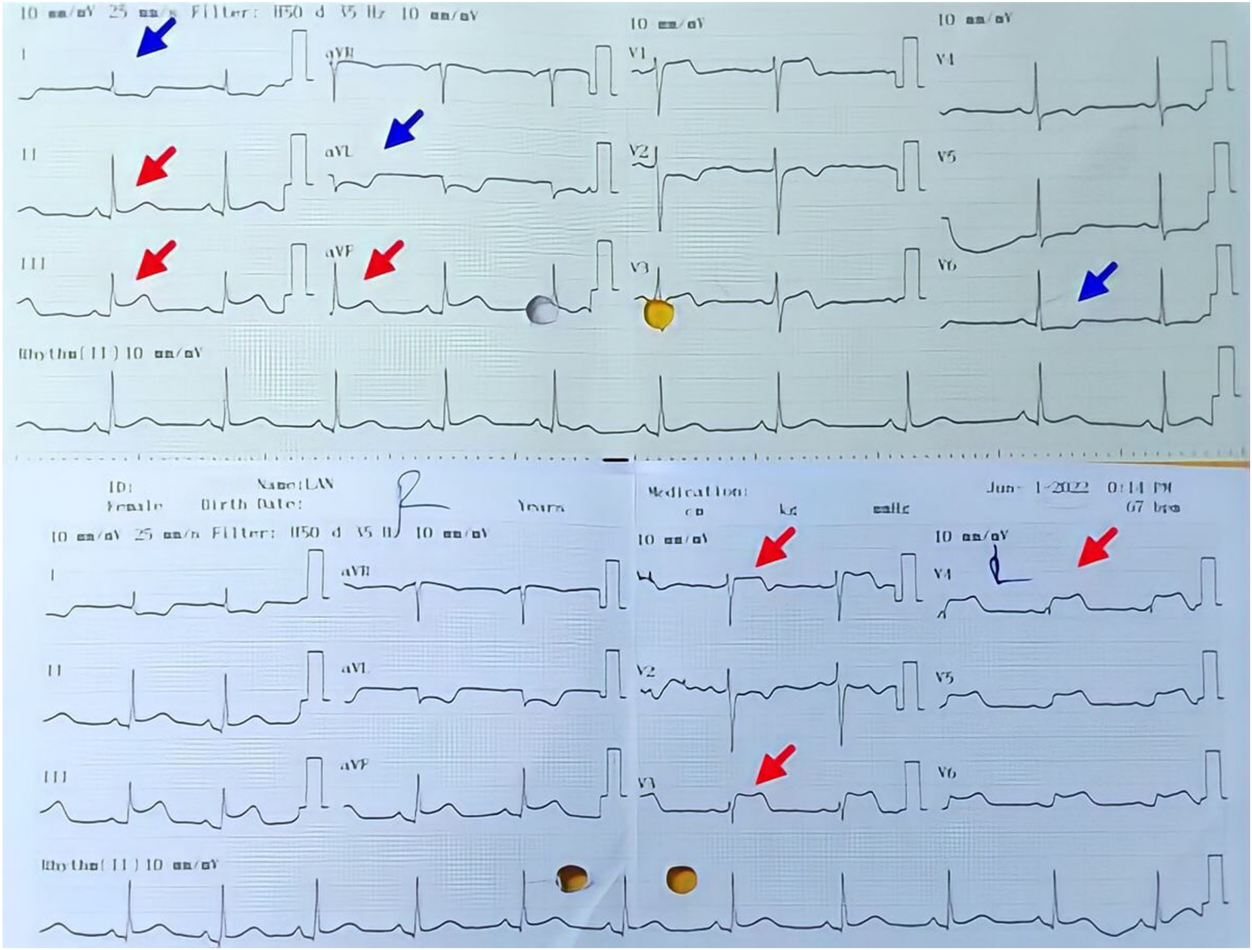
The upper ECG shows a 2 mm ST elevation (red arrow) at leads DII, DIII, aVF, V1, with reciprocal depression (blue arrow) in DI, aVL, and V6. This ECG suggests an inferior myocardial infarction with ST elevation. The lower ECG with right-side lead indicates a 2 mm ST elevation (red arrow) at lead V3R, and V4R which suggests a right ventricular myocardial infarction. RCA was suspected as culprit lesion.

**Figure 2:**
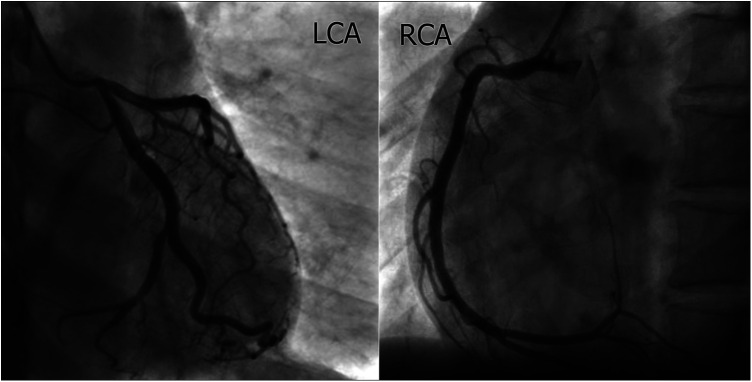
Coronary angiography revealed patent coronaries including LCA (left) and RCA (right) without significant flow-limiting lesions.

**Figure 3:**
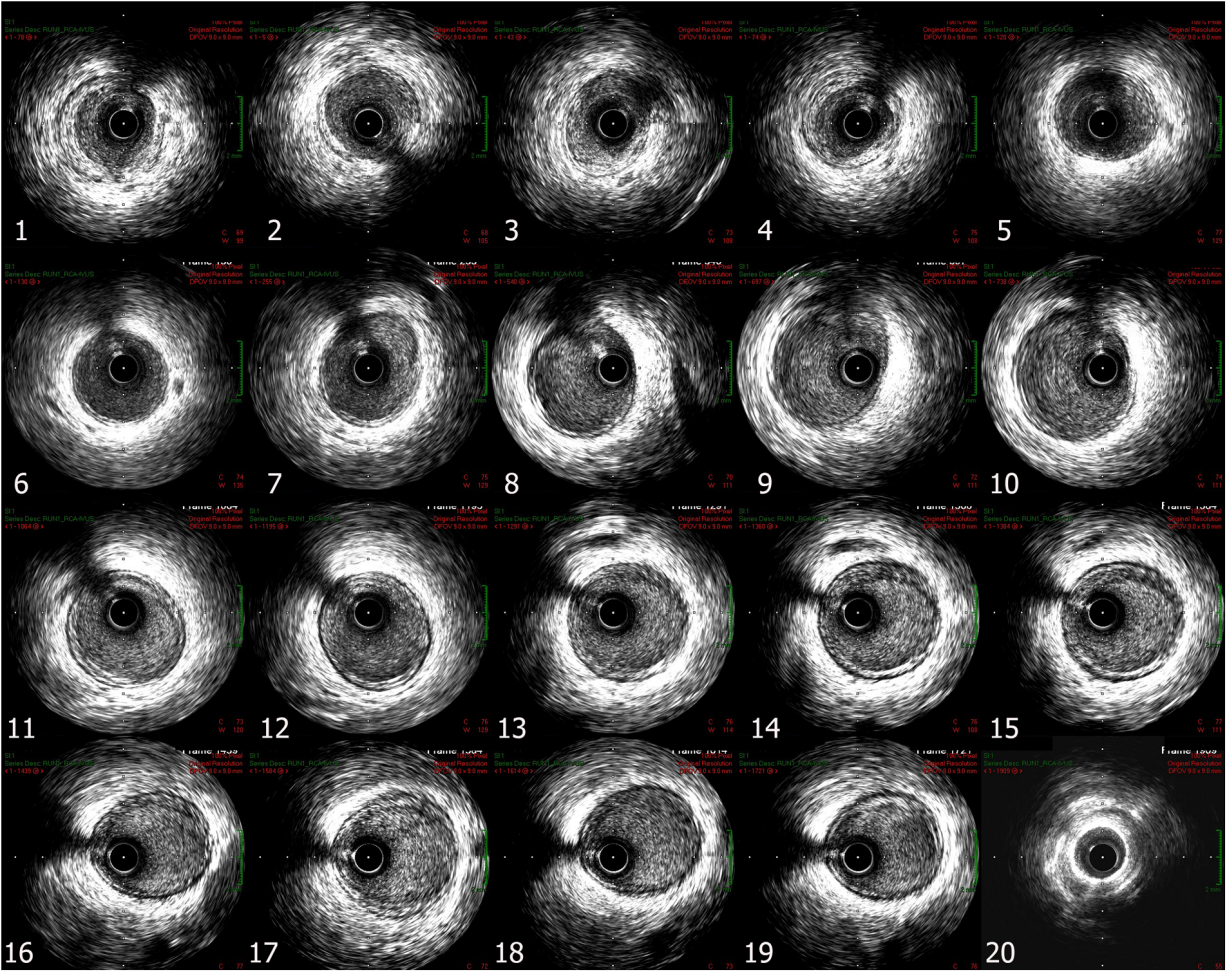
Right coronary artery IVUS showed no significant stenotic lesion, no thrombus, and no dissection in proximal RCA (image 1 to 5), mid RCA (image 6 to 10), and distal RCA (image 11 to 20).

A 42-year-old Vietnamese female (gravida 5 para 3) with a past medical history of hypertension reported taking Mifepristone 200 mg to induce the termination of her 5-week pregnancy one day before admission. The patient continued to take misoprostol 600 mcg sublingually one hour before hospitalization. She then experienced acute severe left-sided chest pain, dyspnea, and nausea prompted her to go to the hospital. Upon arrival, the patient noted bradycardia with a heart rate of 55 beats per minute and hypotension with a blood pressure of 70/40 mmHg. The other vital signs were stable, with a respiratory rate of 20 breaths per minute and saturation at 99% on room air. ECG showed 2 mm ST elevations in II, III, aVF, V1, V3R, and V4R with reciprocal depressions in I, aVL, and V6 (Figure 1). Laboratory findings were remarkable for markedly elevated hs-troponin T (103 ng/l). Since the presentation suggested acute ST-elevation myocardial infarction (Killip I) of the inferior and right ventricle, the patient was started on loading dual anti-platelet therapy and statin. Thirty minutes later, she reported that her chest pain had improved. Repeat ECG showed normal sinus rhythm with a heart rate of 70 beats per minute and complete resolution of ST-elevation. The subsequent coronary angiography revealed normal coronary without flow-limiting lesions (Figure 2). IVUS of RCA showed no evidence of significant stenotic lesions, thrombus, or dissections (Figure 3). Transthoracic echocardiography revealed a preserved ejection fraction of 68% without wall motion abnormalities or other structural heart diseases. Troponin level also decreased to 74 ng/L after 3 h of hospitalization and normalized after six days. The patient satisfied the criteria of MINOCA following the Fourth Universal Definition of Acute Myocardial Infarction with the elevated dynamic change of cardiac biomarker, ST elevation on ECG, and the result of coronary angiography without significant obstructing lesion (6). Due to the correspondence between the pharmacologic characteristics of Misoprostol and the clinical manifestation of patient, we assume the MINOCA is possibly caused by coronary vasospasm induced by misoprostol (6). She was eventually discharged with calcium channel blocker, beta-blocker, and statin. The patient remained asymptomatic and hemodynamically stable during her follow-ups in 30 days, 3 months, 6 months, and 12 months after discharge.

## Discussion

About 6% of patient with acute myocardial infarction is diagnosed with nonobstructive coronary arteries by coronary angiography (7). Myocardial infarction with nonobstructive coronary arteries (MINOCA) is considerably confirmed when there is no evidence of other etiologies at the time of coronary angiography. A definite diagnosis usually requires extensive workup for various pathologies. Montone et al. reported 46% of patients with MINOCA responded to provocation testing, confirming coronary vasospasm (8). Coronary vasospasm is defined as an intense constriction of coronary arteries resulting in a significant imbalance between oxygen demand and supply. This phenomenon can provoke severe myocardial ischemia, acute myocardial infarction, or sudden cardiac death (1). The incidence of this disease varies significantly among races and between countries such as Japanese (24.3%), Taiwanese (19.3%) and Caucasian populations (7.5%) (9). Dr. Myron Prinzmetal first described this condition in 1959 and reported 32 cases of vasospastic angina (non-exertional chest pain) (1). The classic diagnostic criteria require a normal coronary angiography with spasm response to a provocative test (10). Various pathologic mechanisms were proposed, including the direct effect of catecholamines, inflammation, dysfunction of endothelial cells, smooth muscle cell hypercontractility, or increased oxidative stress (10). Compared to classic angina due to atherosclerotic artery disease, vasospastic angina induced by coronary vasospasm frequently happens in young female patients without significant cardiovascular risk factors (11). Moreover, typical precipitating factors for this condition include cold exposure, mental stress, stimulants, and medications, such as sympathomimetics and vasoconstrictor agents (10, 11). The cardiovascular events or adverse effects of misoprostol were summarized in Table 1 (5, 12–21).

**Table 1:** Literature review of previous studies.

Author	Age (year)	Background	Current medications	Indication	Dosage and route	Time	Events	Angiogram
Sung/Korea/2009 (12)	44	Hypertension, hemodialysis	Carvedilol nifedipine valsartan	Abortion	Misoprostol vaginal 1,000 m cg in total in 20 h then IV sulprostone 42 mcg/h	8 hours after sulprostone	Myocardial infarction	RCA narrow responsive to NTG 400 mcg
Miriam/Spain/2010 (13)	32	Primigravida active smoking obesity (BMI 32 kg/m^2^)	No	Termination of pregnancy (17 weeks)	200 mg mifepristone follow with 800 mcg misoprostol vaginally after 48 hours	2 hours	Hypotension, anteroseptal MI	–
Ray/India/2011 (13)	32	Pregnancy-induced hypertension	Methyldopa 250 mg twice daily	Postoperative vaginal bleeding	Misoprostol 800 mcg vaginally	20 minutes	Pulmonary edema	–
Owusu/USA/2015 (15)	42	Hypertension	Norethindrone 0,35 mg daily	Facilitate hysteroscopy	Misoprostol 200 mcg sublingual	–	VF, Inferior and lateral MI, cardiac arrest	Normal left main coronary artery, 60% LAD stenosis 70% RCA stenosis
Prashanth^a^/India/2016 (16)	32	BMI 28 kg/m^2^	–	Facilitate hysteroscopy	Misoprostol vaginally 200 mcg 30 minutes prior to procedure and Misoprostol 600 mcg rectally during the procedure due to blood loss (800 ml)	1 hour	Hypertensive crisis	–
Prashanth^b^/India/2016 (16)	24	Primigravida	–	postpartum hemorrhage	Misoprostol rectally 600 mcg then 250 mcg prostaglandin F2 alpha + and 0,2 mg IV methylergometrine	1 hour 15 min	Hypertensive crisis	–
Matthesen/Denmark/2017 (17)	41	G3P2 One spontaneous abortion One vaginal delivery	–	Termination of pregnancy	Misoprostol (dosage not listed)	Not listed	Cardiac arrest	Coronary angiography revealed coronary artery spasm, which responded to NTG
Mazhar/Saudi Arabia /2018 (**5**)	39	G3P2 One spontaneous abortion	No	Incomplete abortion (6w4d)	Misoprostol 400mcg every 12 h (800 mcg in total)	45 minutes after the second dose	MI	Perform on the fifth day did not yield abnormalities
Munoz-Franco 2019 (18)	10	G6P2 Three miscarriages, active smoking, hyperlipidemia	No	Incomplete abortion	Misoprostol 400 mcg vaginally	20 minutes	Lateral and anterior MI	Severe stenosis in the middle segment of LAD, which responded to NTG
Sugito 2020 (19)	39	Depression	Recent amphetamine use	Termination of pregnancy	Misoprostol (dosage not listed)	1 hour	VF, MI, cardiac arrest	Diffuse multi-vessel coronary spasm, which improved with intracoronary NTG
Lokhande 2021 (20)	57	COVID-19 infection		Uterine bleeding	Misoprostol (dosage not listed)	Not listed	ST elevation in inferior leads	70% stenosis of RCA, which resolved entirely with intracoronary NTG

IV, intravenous, RCA, right coronary artery, NTG, nitroglycerin, MI, myocardial infarction, LAD, left anterior descending, VF, ventricular fibrillation.

Note: a, b: Prashanth et al. reported 2 cases in this paper.

Prostaglandin E (PGE) analogs have both vasoconstrictor and vasodilator properties (Supplementary Image S1). This medication has a long history of adverse cardiovascular effects. PGE2 analog, such as Sulprostone, has been reported to have severe cardiovascular complications such as acute myocardial infarction, cerebral ischemic stroke, and severe hypotension (18, 22, 23). Among the PGE1 analog, misoprostol in combination with mifepristone was approved by the European Medicines Agency for treating incomplete abortion and miscarriage, while Gemeprost was used with caution due to the risk of severe adverse cardiac events (5). The cornerstone mechanism of these complications was the various effect of Prostaglandin E receptors (EP), including EP 1, 2, 3 and 4. While EP 1 and EP 3 induce vasoconstrictors, EP 2 and EP 4 have substantial vasodilator properties. PGE2 analog stimulates all 4 four receptors, while the PGE1 analog activates only EP 2, 3 and 4 (18).

The selective activation of PGE1 explains these agents’ less frequent and severe cardiovascular complications. According to the prevailing hypothesis, it is widely believed that misoprostol exhibits a dose-dependent effect on the elevation of Norepinephrine (NE) levels. Consequently, the increased NE levels are thought to induce pronounced vasoconstriction and contribute to the occurrence of cardiovascular adverse events (24). In patients with medical history or high risk of cardiac disease, misoprostol was recommended to be used at a very low dose of 25 mcg every 4 h in combination with prior Mifepristone 200 mg to limit cardiovascular complications for induction labor (26).

Despite being commonly used in obstetrics and gynecology practice for labor induction and considered generally safe, misoprostol still carries a potential risk of cardiac adverse effects. In this particular patient, the Naranjo Score yields a score of 7 out of 13 points, suggesting a moderate likelihood of misoprostol being responsible for the observed cardiac adverse events (5, 22, 23). On the other hand, misoprostol at higher doses is typically used for terminating a pregnancy, yet evidence of its safety is still unclear. WHO recommends that a single dose of misoprostol 400 mcg should be given orally within 24–48 h after taking mifepristone to induce medical abortion for less than 7-week pregnancy. Additionally, besides the dose, the route of administration can impact the process of absorption, bioavailability, and the concentration of active compounds in the bloodstream (27). Misoprostol is usually administered by several pathways, including buccal, sublingual, vaginal, oral, or rectal. Among various routes, a sublingual pathway has the highest peak of plasma drug concentration while the vaginal with water has the most prolonged time of adequate drug concentration (25). So theoretically, the risk of adverse events is highest in these two administration pathways.

Thus far, there are no definitive criteria to diagnose Misoprostol-induced coronary spasm. In our case, the patient with risk factors for coronary artery disease exposed to Misoprostol 15–20 minutes before the onset of chest pain suggested a probability of association. Additionally, the plasma concentration of Misoprostol peaks at about 30 min and declines rapidly by 120 min if used orally (25). This pharmacokinetic can explain the resolution of chest pain and normalization of ST-T changes on ECG shortly after hospitalization. Moreover, the diagnosis is more consistent with coronary vasospasm when the following angiogram and IVUS exclude occluded coronary arteries. The dynamic changes of cardiac biomarkers, ECG and clinical symptoms of the patient suggest the diagnosis of MINOCA possibly due to coronary vasospasm induced by misoprostol. New imaging modalities such as optical coherence tomography and cardiac magnetic resonance (CMR) can be used to clarify the underlying pathologic mechanism. CMR plays a crucial role in the diagnostic evaluation of patients with MINOCA, as it is recommended for excluding non-ischemic cardiac causes including cardiomyopathies, myocarditis, pericarditis, and Takotsubo syndrome. CMR has been found to be useful in confirming the diagnosis in up to 74% of cases (6). However, further investigation of this case is challenging due to our limited facilities.

## Conclusion

Myocardial infarction with nonobstructive coronary arteries due to acute coronary vasospasm is a severe condition precipitated by medications with vasoconstricting properties, such as misoprostol, as a PGE1 analog. Notwithstanding isolated case reports describing notable cardiovascular events linked to misoprostol administration, it is considered a safe and appropriate choice as the initial treatment option for medical abortion, aligning with the recommendations outlined in the current practice guidelines.

Our case proposes a precaution in using misoprostol in patients with a high risk for cardiovascular disease, who should be closely monitored for any emerging severe complications.

## Data Availability

The original contributions presented in the study are included in the article/**Supplementary Material**, further inquiries can be directed to the corresponding author.
