# Supplementary figures and images for: A case report and literature review of myocardial infarction with nonobstructive coronary arteries (MINOCA) possibly due to acute coronary vasospasm induced by misoprostol
Source: Front Cardiovasc Med. 2023 May 26;10:1115358. doi: 10.3389/fcvm.2023.1115358 (PMC10250731; doi:10.3389/fcvm.2023.1115358)

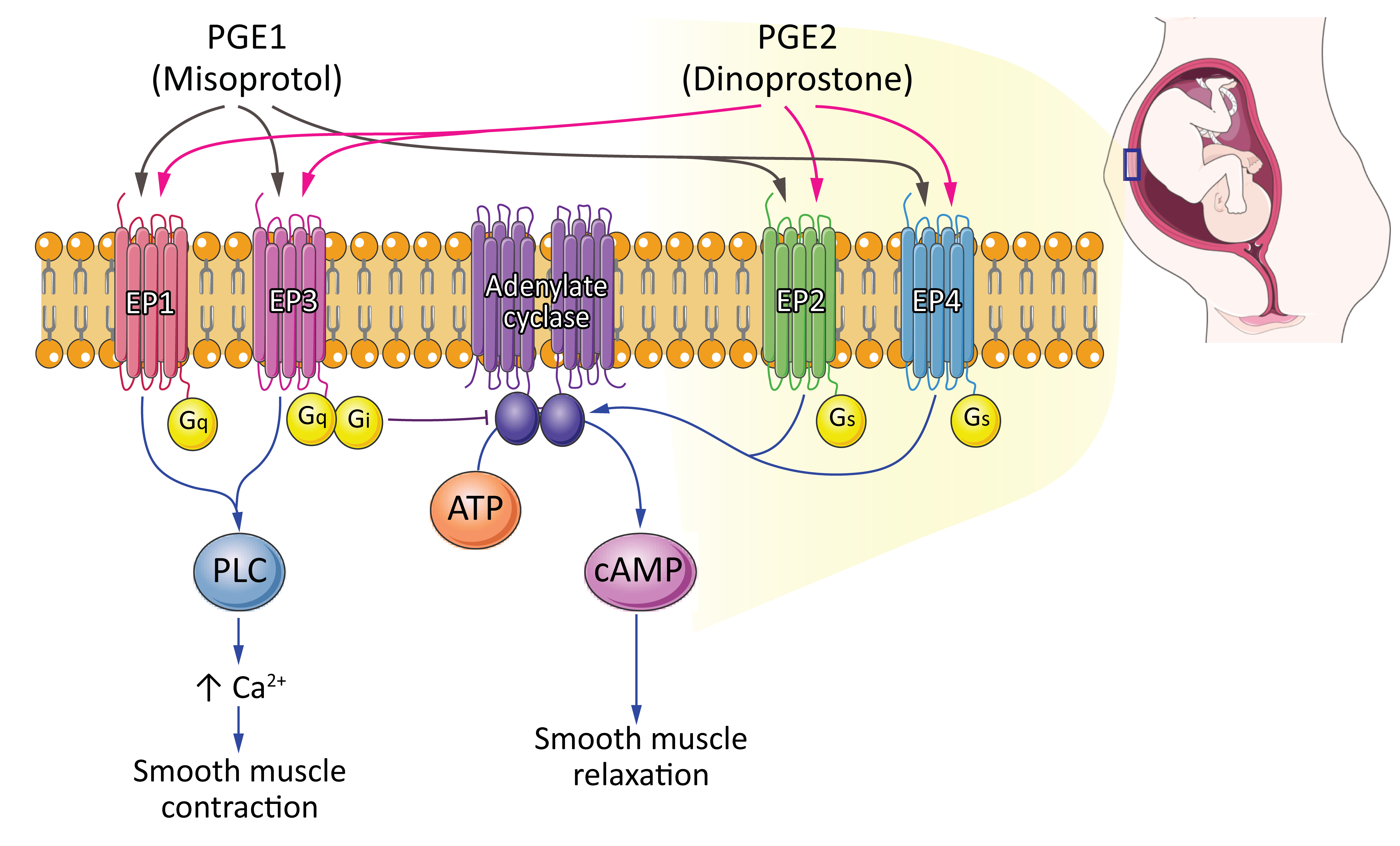

Supplement: Supplementary file 1 [file Image1.jpeg]
